# Models of risk prediction for lung infections following a stroke: A systematic review

**DOI:** 10.1097/MD.0000000000047646

**Published:** 2026-02-20

**Authors:** Ning Dong, Yuan-Yue Pang, Yu-Ying Li, Qiu-Yan Liu, Xue Dong

**Affiliations:** a Qian Wei Hospital of Ji Lin Province, Changchun, China; b Department of Nursing, Changchun University of Chinese Medicine, Changchun, China.

**Keywords:** prediction, pulmonary infection, risk factors, stroke, system review

## Abstract

**Background:**

Stroke is the leading cause of disability and second leading cause of death worldwide. Pulmonay infection represents the most fatal complication following stoke, accounting for substantial mortality and economic burden. Although various prediction models have been developed to identify susceptible patients, systematic evaluation of their clinical utility to guide therapeutic management is required. In order to use the models that forecast risks for lung infections among patients with stroke as a reference for therapeutic management, this study aims to systematically evaluate them.

**Methods:**

Relevant materials were found by searching databases such as the Cochrane Library, PubMed, MEDLINE, Embase, Wanfang, VIP, CBM, and China National Knowledge Infrastructure. Studies on risk prediction models for poststroke pulmonary infections that were published from database’s creation to December 2023 were included in the search. The included studies were analyzed and compared in terms of characteristics, research types, predictive factors, model construction methods, and results.

**Results:**

Twenty-four models from 16 studies were included, and the included models’ area under the curve values from 0.740 to 0.960, and there were 8 to 32 possible predictors. Age, National Institutes of Health Stroke Scale score, dysphagia, diabetes history, and disturbance of consciousness (Glasgow Coma Scale score) were the most frequently reported predictors. Because of the unreasonably large sample size, the single-factor analysis used to screen predictive features, and the absence of a model performance evaluation, the models demonstrated had not showed a high risk of bias.

**Conclusion:**

Predictive algorithms for the risk of lung infections in patients with stroke are still being studied. Prospective studies should focus on improving research design and reporting, and conducting internal and external validation to develop localized, high-performance, and user-friendly predictive models.

## 1. Introduction

Nowadays, stroke is the most prevalent cause of disability and the second leading cause of death worldwide. As the world’s population ages more quickly, the incidence of stroke is also steadily rising.^[1]^ In particular, pulmonary infections have the highest proportion of deaths among stroke-related infections, accounting for about one-third of the total deaths.^[2]^ Moreover, it is a serious economic burden for family and society that 459 million dollars are spent on curing infections related to stroke every year.^[3]^ Therefore, How to prevent effectively stroke patient from lung infection is extremely essential to improve prognosis. It is recommended that Physicians can more precisely determine the likelihood or risk of pulmonary infection in the future by using predictive models, with giving them a scientific foundation for creating individualized treatment plans and enhancing patient outcomes.

At present, multiple domestic and international studies have investigated the factors that predict pulmonary infection in the stroke patients. Some of these studies have accurately measured the likelihood of pulmonary infection by developing prediction models. The predictors within the model are easy to assess with faster and more convenient using. This paper is looking to present a reference basis for reducing the incidence of following a stroke infection in clinical practice, which analyzes the risk factors of pulmonary infection in stroke patients and evaluates systematically the risk prediction model for patients with poststroke complications of pulmonary infection.

## 2. Methods

### 2.1. Study design

The object of this study is to assess the risk prediction model for pulmonary infection in stroke patients through a systematic review. The Preferred Reporting Items for Systematic Reviews standards, which are listed in Table 1, are followed in this review. This study was not prospectively registered on a publicly accessible registry such as PROSPERO. Ethics approval was not required, because this systematic review used publicly published data only.

**Table 1:** Basic characteristics of included studies on pulmonary infection risk prediction models in stroke patients.

Study	Publication year	Country	Study population	Study design	Assessment tool
He et al^[4]^	2019	China	Acute stroke patients	Retrospective	Hospital infection diagnosis standard
Huang et al^[5]^	2019	China	Acute ischemic stroke patients	Prospective	CDC
Yang et al^[6]^	2021	China	Stroke patients	Retrospective	Pulmonary ultrasound examination combined with plasma procalcitonin and C-reactive protein detection
Zhang et al^[7]^	2021	China	Stroke patients	Retrospective	–
Wang et al^[8]^	2022	China	Stroke patients	Retrospective	Diagnosis standard for pulmonary MDRB infection
Fang et al^[9]^	2023	China	Acute stroke patients	Retrospective	–
Yan et al^[10]^	2022	China	Intracerebral hemorrhage patients	Retrospective	PISCES
Zhang et al^[11]^	2023	China	Acute ischemic stroke patients	Retrospective	CDC
Sarah et al^[12]^	2012	Germany	Ischemic stroke patients	Prospective	CDC
Ji et al^[13]^	2013	China	Acute ischemic stroke patients	Prospective	CDC
Ji et al^[14]^	2014	China	Spontaneous intracerebral hemorrhage patients	Prospective	CDC
Ge et al^[15]^	2019	China	Acute ischemic stroke patients	Retrospective	2010 Chinese expert consensus on diagnosis and treatment of stroke-related pneumonia
Li et al^[16]^	2020	China	Acute ischemicstroke patients	Prospective	–
Wang et al^[17]^	2020	China	Acute intracerebral hemorrhage patients	Retrospective	CDC
Wu et al^[18]^	2020	China	Elderly acute intracerebral hemorrhage patients	Retrospective	2019 Updated Chinese expert consensus on diagnosis and treatment of stroke-related pneumonia
Wang et al^[19]^	2021	China	Acute ischemic stroke patients	Retrospective	2010 Chinese expert consensus on diagnosis and treatment of stroke-related pneumonia

CDC: US Centers for Disease Control and Prevention; PISCES: pneumonia in stroke patients, a diagnostic tool from the stroke consensus group; MDRB: multidrug-resistant bacteria; “‐”: not reported.

### 2.2. Search strategy

We searched a number of databases, including Cnki, Wanfang Data Knowledge Service Platform, VIP, CBM, PubMed, Embase, MEDLINE, and the Cochrane Library, in-depth and thoroughly for literature on risk prediction models for pulmonary infection following stroke. Subject words and key words were used to search. This was supplemented by manual searching of published and traceable literature. The time frame for the search was from the database’s creation to December 2023.

The Chinese database that Wanfang database as an example, and the search formula was as follows. Subject: (stroke OR stroke OR cerebrovascular accident) and subject: (pulmonary infection OR pneumonia) and subject: (prediction OR warning OR risk score OR risk assessment) and subject: (model OR tool). English databases took PubMed as an example, the search formula is (“stroke” [MeSH]) AND (“pulmonary infection” [All Fields] OR “penumonia” [All Fields]) AND (predict*[All Fields] OR“risk prediction” [All Fields] OR“risk score” [All Fields] OR “risk assessment” [All Fields]) AND (“model” [All Fields] OR “score” [All Fields]).

### 2.3. Study selection and eligibility criteria

Two reviewers independently filtered the search results by looking at abstracts and titles. Then, they thoroughly examined the full-text publications to ascertain their applicability. When there was a dispute, a third reviewer attended to offer a different viewpoint. The inclusion criteria were: The study subjects were patients aged ≥18 years with a diagnosis of stroke confirmed by CT or MRI; The study was a model construction and/or validation study for predicting the risk of concomitant pulmonary infections after stroke; The type of study was a cohort or case-control study and a cross-sectional study; and The language of the article was Chinese or English.

Exclusion criteria included studies that only analyzed risk factors or predictors of pulmonary infection after stroke without establishing a prediction model, studies where the full text was unavailable, duplicated literature, and conference abstracts, academic papers, and other informally published documents.

### 2.4. Data extraction

In our study, 2 researchers strictly followed the inclusion and exclusion criteria to independently screen the literature, and used EndNote literature management software to de-duplicate the obtained literature. A third researcher was invited to make judgments on controversial literature. The quality of case-control and cohort studies was assessed using the Newcastle–Ottawa Scale.

### 2.5. Model bias risk assessment

Four fields were evaluated for bias risk: analysis, results, predictors, and study participants. All of the 20 inquiries in these 4 domains could be answered with “yes,” “probably yes,” “no,” “probably no,” or “no information.” The bias risk for a domain was deemed low if every question received a “yes” or “probably yes” response. If any question was answered with “no” or “probably no,” the bias risk for that domain was considered high. If there was a lack of relevant information, the bias risk for that domain was regarded as unclear. The study’s general bias risk was deemed low if it was low in each of the domains. The total chance of bias was deemed high if the bias risk was high in any one of the domains. If the bias risk was unclear in any domain while it was low in the others, the overall bias risk of the study was considered unclear.

### 2.6. Assessment of applicability

The assessment of applicability covered 3 domains: study participants, predictors, and outcomes. Each domain was evaluated as having “good,” “poor,” or “unclear” applicability. If all domains were rated as having good applicability, the overall applicability of the study was considered good. If any domain was rated as having poor applicability, the overall applicability was deemed poor. If any domain was rated as having unclear applicability while the others were rated as good, the overall applicability of the study was considered unclear.

## 3. Results

**Figure 1.**
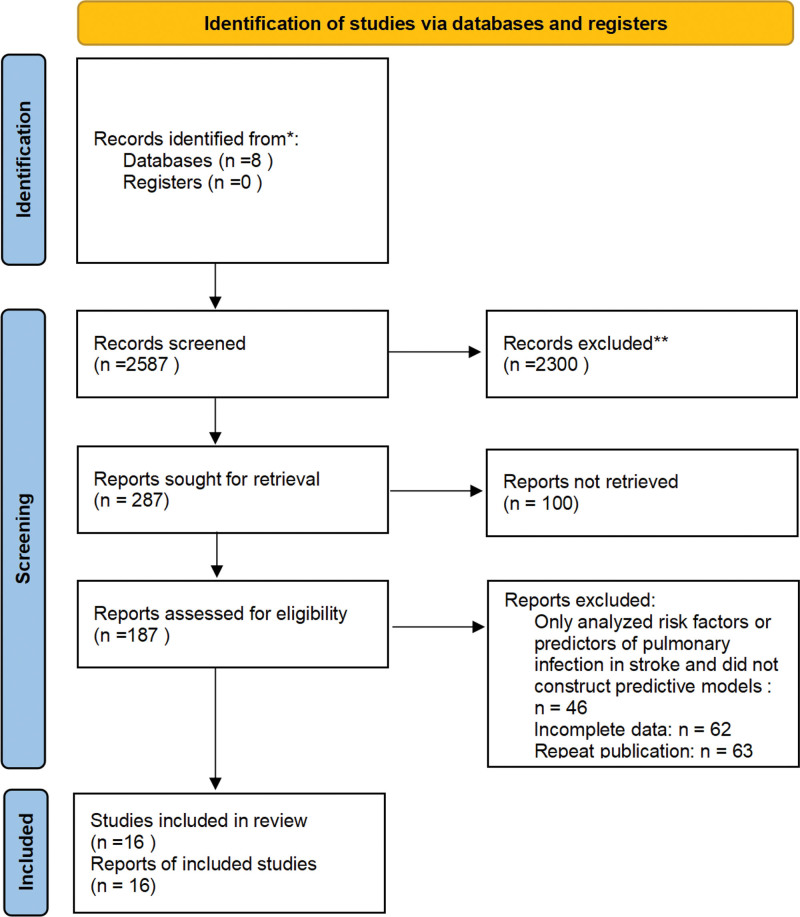
Retrieval and screening process flowchart. This diagram illustrates the systematic review process using PRISMA 2020 guidelines. Arrows indicate the flow of records from identification to inclusion. 8 databases yielded 2587 records, of which 2300 were excluded after screening. 287 full reports were retrieved, and 187 were assessed for eligibility. 16 studies were included after excluding 171 due to various criteria. Arrows denote the progression and reasons for exclusion at each stage.

In this study, 2587 pertinent articles were found totally. Eventually, 16 articles^[4–19]^ with 24 prediction models were included after first screening titles and then screening keywords. Detailed processes are provided in the form of PRISMA flowcharts (Fig. 1).

### 3.1. Basic characteristics

Among the 16 articles, 1 study was conducted in European and American countries, and 15 studies were conducted in Asian countries. Of the 16 studies, 5^[5,12–14,16]^ were prospective cohort studies, and 11^[4,6–11,15,17–19]^ were retrospective cohort studies. Additionally, the included article’s predictive outcomes were all the occurrence of pulmonary infection after stroke. The basic characteristics of the included articles are shown in Table 1.

### 3.2. The establishment of the risk prediction models

In the 24 prediction models included, 16 of them^[4–19]^ were dedicated to model development and validation studies. The total sample size across the various models ranged from 100 to 83,063 cases, and the number of outcome events varied from 40 to 705,400 cases. Regarding the selection of predictive variables, 12 studies^[4,5,7–9,11–14,17–19]^ screened predictive factors based on univariate analysis, with selecting those statistical significance (*P* < .05) without combining them with other independent variables for analysis. Details of the model establishment are presented in Table 2.

**Table 2:** Establishment of pulmonary infection risk prediction models in stroke patients.

Study	Predictors		Total sample size			Missing data	Whether to avoid univariate analysis to screen predictors
	Number of potential predictors (n)	Number of final predictor (n)	Overall sample size (n)	Modeling sample size (n)	Sample size of test samples (n)	Processing method	
He et al^[4]^	19	6	433	40	393	–	N
Huang et al^[5]^	12	7	983	643	340	–	N
Yang et al^[6]^	13	6	230	66	164	–	Y
Zhang et al^[7]^	10	7	176	40	136	–	N
Wang et al^[8]^	16	5	100	73	27	–	N
Fang et al^[9]^	19	7	440	440	188	–	N
Yan et al^[10]^	12	9	83,063	70,540	12,523	Multiple imputation	N
Zhang et al^[11]^	27	4	400	295	105	–	N
Sarah et al^[12]^	8	5	15,335	15,335	45,085	Delete	N
Ji et al^[13]^	27	11	14,702	8820	5882	–	N
Ji et al^[14]^	32	11	4998	2998	2000	–	N
Ge et al^[15]^	–	25	13,930	11,736	2194	Delete	N
Li et al^[16]^	18	6	3160	2528	632	Delete	Y
Wang et al^[17]^	16	4	2303	1841	462	–	N
Wu et al^[18]^	17	7	173	173	–	–	N
Wang et al^[19]^	17	8	257	257	–	–	N

“‐” indicates not reported.

### 3.3. Model performance and predictor variables

Every included study^[4–19]^ provided the area under the curve (AUC) of the receiver operating characteristic curve. Among them, 11 studies^[4,5,7,8,10,12–14,16,18,19]^ reported the credible interval of the AUC, and 11 studies demonstrated good discriminative ability (AUC ≥ 0.80). Goodness-of-fit tests were performed on 2 models,^[8,9]^ and both had *P* ≥ .05, suggesting a robust consistency. The models included in these studies comprised 8 to 32 potential predictive variables, with age being the most frequently occurring predictive variable. In terms of model validation, 14 researches^[4–10,13–19]^ included internal evaluation as well as model creation. The model establishment details were displayed in Table 3.

**Table 3:** Performance of lung infection risk prediction models and predictors in stroke patients.

Study	Model performance		Whether it is verified internally	Predictors included at the end
	AUC	Calibration method		
He et al^[4]^	Modeling: 0.90 (0.849–0.964); Validation: –	Calibration curve	Y	Stroke type, NIHSS score, dysphagia, white blood cell count, renal dysfunction (blood creatinine), history of diabetes
Huang et al^[5]^	Modeling: 0.845 (95% CI 0.814–0.872); Validation: 0.897 (95% CI 0.860–0.927)	Calibration curve	Y	Age, NIHSS score, nasogastric tube intervention, mechanical ventilation, Atrial Fibrillation
Yang et al^[6]^	0.747	–	Y	Smoking history, immune dysfunction, stroke type, NIHSS score, nasogastric tube, respiratory system disease
Zhang et al^[7]^	0.918 (0.868–0.968) ; Validation: –	–	Y	Age, stroke type, diabetes history, smoking history, dysphagia, consciousness disturbance, invasive operations
Wang et al^[8]^	0.924 (0.865–0.983)	Goodness-of-fit test *P* = .598	Y	History of diabetes, prophylactic use of antibiotics, consciousness disturbance, invasive procedures, ICU stay ≥ 72 hours
Fang et al^[9]^	Modeling: 0.931; Validation: 0.927	Goodness-of-fit test *P* = .49	Y	NIHSS score, invasive operations, elevated CRP and PCT, hypoproteinemia, oral dysfunction
Yan et al^[10]^	Modeling: 0.749 (0.739, 0.759); Validation: 0.784 (0.774, 0.794)	Calibration curve	Y	Age, mRS, fasting blood glucose, NIHSS score, GCS score, CRP, dysphagia, COPD, current smoking
Zhang et al^[11]^	Modeling: 0.951; Validation: 0.946	Calibration curve	Y	Age, NLR, NIHSS score, V–VST
Sarah et al^[12]^	Modeling: 0.837 (0.826, 0.849); Validation: 0.835 (0.828, 0.842)	Calibration curve	Y	Age, sex, atrial fibrillation, NIHSS score, dysphagia
Ji et al^[13]^	0.797 (0.782, 0.811); Modeling: 0.785 (0.766, 0.803); Validation: 0.792 (0.761, 0.823)	Hosmer–Lemeshow test, calibration curve	Y	Age, history of atrial fibrillation, chronic heart failure, chronic obstructive pulmonary disease, current smoking, mRS score, dysphagia, admission NIHSS score, GCS score, OCSP, blood glucose
Ji et al^[14]^	Modeling: ICH-APS-A model: 0.750 (0.720, 0.770); ICH-APS-B model: 0.740 (0.710, 0.760); Validation: ICH-APS-A model: 0.760 (0.710, 0.790); ICH-APS-B model: 0.740 (0.710, 0.76)	Hosmer–Lemeshow test, goodness-of-fit test, calibration curve	Y	Age, current smoking, excessive alcohol consumption, COPD, NIHSS score, GCS score, dysphagia, location of intracerebral hemorrhage, mRS score, hemorrhage volume
Ge et al^[15]^	Modeling: 0.960; Validation: 0.928	–	Y	Proton pump inhibitors, heparin sodium, Rh(D) blood type, lactate dehydrogenase, ferritin, torasemide injection, pressure ulcer risk factors, neutrophil percentage, history of hypertension, consciousness disturbance, erythrocyte sedimentation rate, invasive operations, age, alanine glutamine injection, omeprazole injection, troponin, donepezil hydrochloride, CRP, nitroprusside injection, short peptide enteral nutrition, dysphagia, furosemide, B-type natriuretic peptide, fall risk assessment scale, mannitol
Li et al^[16]^	Modeling: 0.841 (0.801, 0.881); Validation: –	–	Y	Age, sex, admission NIHSS score, mRS score, atrial fibrillation, fasting blood glucose
Wang et al^[17]^	Modeling: model 1 (logistic regression): 0.778; model 2 (CatBoost): 0.758; model 3 (XGBoost): 0.844; model 4 (LightGBM) : 0.822; Validation: model 1 (logistic regression): 0.776; model 2 (CatBoost): 0.692; model 3 (XGBoost): 0.736; model 4 (LightGBM): 0.767	–	Y	Age, dysphagia, NIHSS score, white blood cell count
Wu et al^[18]^	Modeling: 0.843 (0.811, 0.875); Validation: –	Calibration curve	Y	Age, underlying lung disease, dysphagia, admission NIHSS score, invasive airway operations, nasogastric feeding, prophylactic use of acid suppressants
Wang et al^[19]^	Modeling: 0.860 (0.829, 0.891); Validation: –	Calibration curve	Y	Dysphagia, consciousness disturbance, history of diabetes, nasogastric feeding, prophylactic use of antibiotics, prophylactic use of gastric mucosal protectants, admission NIHSS score ≥ 10 points, age ≥ 65 years

AUC = area under the curve, CRP = C-reactive protein, GCS score = Glasgow Coma Scale score, mRS score = modified Rankin Scale score, NIHSS score = National Institutes of Health Stroke Scale score, NLR = neutrophil-to-lymphocyte ratio, OCSP = Oxfordshire Community Stroke Project, PCT = procalcitonin, V–VST = volume–viscosity swallowing test.

### 3.4. Bias risk and applicability assessment results

In the domain of bias risk intensity regarding the research objects, 10 studies were rated as having a high risk of bias.^[4,6–12,16,17]^ The reasons are that retrospective studies may be subject to recall bias or misclassification bias of outcome events. Moreover, some important predictive factors associated with pulmonary infection after stroke may not be available in medical records, or the assessors were not uniformly trained, leading to biased assessment results. In the domain of predictive factors, all studies were rated as having a low risk of bias for the following reasons: among the 16 included studies, the researchers provided detailed descriptions of the measurement methods for predictive factors, and the answer to the item “Is the definition and assessment of predictive factors the same for all research objects” was “probably yes.” In terms of applicability, all the included models had good applicability in all domains and overall. Table 4 displayed the specifics of the applicability of all the included models in all domains and overall. In addition to the methodological limitations mentioned above, existing model studies generally lack exploration of the pathophysiological or clinical mechanisms underlying key predictive factors, which affects the biological rationality of the models and the identification of potential intervention targets. Taking the history of diabetes, one of the most common predictors in this systematic evaluation, as an example, its association with the significantly increased risk of pulmonary infection after stroke can be explained from multiple perspectives: the hyperglycemic environment directly damages neutrophil chemotaxis, phagocytosis and bactericidal functions, and weakens T lymphocyte response, leading to immunosuppression; Autonomic neuropathy related to diabetes (such as gastroparesis, pharyngeal sensorimotor dysfunction, and weakened cough reflex) significantly increases the risk of aspiration, and has a superimposed effect on the neurological damage caused by stroke itself; The microvascular disease caused by long-term hyperglycemia affects the oxygenation and repair ability of lung tissue; Patients with diabetes are often associated with cardiovascular and renal diseases, which increase the susceptibility to infection; In addition, stress hyperglycemia in the acute stage of stroke is more difficult to control in diabetes patients, which further impairs immune function. Deeply understanding these mechanisms not only strengthens the scientific basis for incorporating the history of diabetes into the model, but also highlights its clinical application value-suggesting that stroke patients with diabetes should implement more stringent blood glucose management, more refined assessment and intervention of swallowing function, and strengthen respiratory tract nursing and other targeted measures, so as to more effectively reduce the risk of lung infection.

**Table 4:** Bias risk and applicability evaluation results of included studies in stroke patients.

Study	Bias risk				Applicability			Overall	
	Study population	Predictors	Outcome	Analysis	Study population	Predictive factors	Outcome	Bias risk	Applicability
Dan et al^[4]^	‐	+	+	?	+	+	+	‐	+
Huang et al^[5]^	+	+	+	?	+	+	+	‐	+
Yang et al^[6]^	‐	+	+	?	+	+	+	‐	+
Zhang et al^[7]^	‐	+	+	?	+	+	+	‐	+
Wang et al^[8]^	‐	+	+	?	+	+	+	‐	+
Fang et al^[9]^	‐	+	+	?	+	+	+	‐	+
Yan et al^[10]^	‐	+	+	?	+	+	+	?	+
Zhang et al^[11]^	‐	+	+	?	+	+	+	‐	+
Sarah et al^[12]^	‐	+	+	?	+	+	+	‐	+
Ji et al^[13]^	+	+	+	?	+	+	+	?	+
Ji et al^[14]^	+	+	+	?	+	+	+	?	+
Ge et al^[15]^	+	+	+	?	+	+	+	‐	+
Li et al^[16]^	‐	+	+	?	+	+	+	‐	+
Wang et al^[17]^	‐	+	+	?	+	+	+	‐	+
Wu et al^[18]^	+	+	+	?	+	+	+	‐	+
Wanget al^[19]^	+	+	+	‐	+	+	+	‐	+

“+” denotes high applicability and low risk of bias; “‐” denotes low applicability and high risk of bias; and “?” denotes ambiguous.

## 4. Discussion

### 4.1. Limitations in the predictive model for pulmonary infection in stroke patients

Patients with stroke are prone to pulmonary infection due to prolonged bed rest and lack of exercise, which leads to the accumulation of respiratory secretions in the lower respiratory tract. Additionally, clinical invasive procedures such as endotracheal intubation and suctioning can easily cause pathogens to linger in the lower respiratory tract, leading to infection.^[20]^ Furthermore, the development of scoring systems to predict the risk of pneumonia following a stroke has been investigated by both domestic and foreign researchers.^[13,14,21]^ Yet, there are significant differences in the evaluation indicators of different scoring systems, like that some scales are not convenient or the individual risk of disease onset is not precise enough.

Currently, the logistic regression model is commonly used in clinical practice for risk factor prediction and analysis, which can reflect the relationship between independent and dependent variables. With the increase in the incidence of pulmonary infection after stroke, the number of risk prediction models is also increasing. It is particularly important to select high-quality models to provide a basis for clinical research and practice. Twelve studies all used univariate analysis to identify variables with statistical significance related to pulmonary infection after stroke, and then included these variables in the regression model for analysis. Twelve studies^[4,6–9,11–14,17–19]^ identified variables related to pulmonary infection after stroke through univariate analysis, which were then incorporated into regression models for further analysis. This screening strategy based on statistical methods can reduce the number of predictors during model development, generating a simpler model.

However, choosing independent variables only through statistical methods could miss important risk factors. It is recommended to use Lasso regularization for variable selection in predictive models because it compresses the coefficients of unimportant variables to zero, achieving efficient feature selection, especially suitable for high-dimensional data. This method not only reduces model complexity and prevents overfitting but also improves generalization ability and enhances model interpretability. Compared with traditional methods (such as stepwise regression), Lasso is more computationally efficient and stable, making it an ideal choice for building concise predictive models. Additionally, we should exercise caution when including and deleting variables, even if they lack statistical significance.^[22]^

Also, given the high number of potential predictors for poststroke pulmonary infection, the sample size may fall short of the recommended standard of 20 events per predictor variable. Among the 16 studies included in this study, only the study by Yan et al^[10]^ used multiple imputation to handle missing data, 3 studies^[12,15,16]^ directly excluded subjects with missing data, and the rest did not report. This approach may lead to significant differences between the excluded subjects and those finally included in the statistical analysis, resulting in bias in the predictive results and model performance.

### 4.2. Choosing the right predictive model for clinical use

The significance of establishing a risk prediction model for lung infection after stroke is to give a risk assessment tool for clinical work, assisting medical professionals to recognize the risks of pulmonary disease in stroke patients as early as feasible. To select an appropriate model, medical staff should comprehensively consider its applicability, predictive performance, availability of predictive parameters, and ease of use before choosing one. All of the 24 prediction models included were developed based on stroke patients. Each model has its strengths, but a model’s discriminative ability is the most crucial factor in assessing performance. If this ability is weak, there’s no point in evaluating other metrics further. Moreover, the number of predictive variables for pulmonary infection after stroke is relatively large, ranging from 8 to 32 in the included literature. In the studies that establish prediction models, researchers collect some predictive factors for statistical analysis. However, the more predictive factors there are, the greater the possibility of overfitting in the prediction model. A streamlined model is easier to apply in clinical practice.^[23]^ Hence, an appropriate model should also be selected.

## 5. Conclusion

In summary, this investigation includes 16 studies with 24 risk prediction models for patients with stroke who have lung infections. The findings demonstrated that there are still certain limitations and a lack of internal and external validation in the research on risk prediction models for pulmonary infection following stroke. Future research should improve the study design and reporting, and conduct validation in different regions and populations.

## Author contributions

**Conceptualization:** Ning Dong.

**Data curation:** Ning Dong.

**Supervision:** Xue Dong.

**Writing – original draft:** Ning Dong, Yuan-yue Pang.

**Writing – review & editing:** Yuan-yue Pang, Yu-ying Li, Qiu-yan Liu.
